# Quantitative Assessment of Hand Spasticity After Stroke: Imaging Correlates and Impact on Motor Recovery

**DOI:** 10.3389/fneur.2019.00836

**Published:** 2019-08-12

**Authors:** Jeanette Plantin, Gaia V. Pennati, Pauline Roca, Jean-Claude Baron, Evaldas Laurencikas, Karin Weber, Alison K. Godbolt, Jörgen Borg, Påvel G. Lindberg

**Affiliations:** ^1^Division of Rehabilitation Medicine, Department of Clinical Sciences, Karolinska Institutet, Danderyd University Hospital, Stockholm, Sweden; ^2^Institut de Psychiatrie et Neurosciences de Paris, Inserm U1266, Paris, France; ^3^Department of Neuroimaging, Sainte-Anne Hospital Center, Université Paris Descartes Sorbonne Paris Cité, Paris, France; ^4^Department of Neurology, Hôpital Sainte-Anne, Université de Paris, Paris, France; ^5^Division of Radiology, Department of Clinical Sciences, Karolinska Institutet, Danderyd University Hospital, Stockholm, Sweden

**Keywords:** observational study, muscle spasticity, stroke rehabilitation, hand, prognosis, magnetic resonance imaging

## Abstract

**Objective:** This longitudinal observational study investigated how neural stretch-resistance in wrist and finger flexors develops after stroke and relates to motor recovery, secondary complications, and lesion location.

**Methods:** Sixty-one patients were assessed at 3 weeks (T1), three (T2), and 6 months (T3) after stroke using the NeuroFlexor method and clinical tests. Magnetic Resonance Imaging was used to calculate weighted corticospinal tract lesion load (wCST-LL) and to perform voxel-based lesion symptom mapping.

**Results:** NeuroFlexor assessment demonstrated spasticity (neural component [NC] >3.4N normative cut-off) in 33% of patients at T1 and in 51% at T3. Four subgroups were identified: early Severe spasticity (*n* = 10), early Moderate spasticity (*n* = 10), Late developing spasticity (*n* = 17) and No spasticity (*n* = 24). All except the Severe spasticity group improved significantly in Fugl-Meyer Assessment (FMA-HAND) to T3. The Severe and Late spasticity groups did not improve in Box and Blocks Test. The Severe spasticity group showed a 25° reduction in passive range of movement and more frequent arm pain at T3. wCST-LL correlated positively with NC at T1 and T3, even after controlling for FMA-HAND and lesion volume. Voxel-based lesion symptom mapping showed that lesioned white matter below cortical hand knob correlated positively with NC.

**Conclusion:** Severe hand spasticity early after stroke is negatively associated with hand motor recovery and positively associated with the development of secondary complications. Corticospinal tract damage predicts development of spasticity. Early quantitative hand spasticity measurement may have potential to predict motor recovery and could guide targeted rehabilitation interventions after stroke.

## Introduction

Spasticity of the muscles contributing to hand function (hereafter “hand spasticity”) is a common sensorimotor disorder after stroke (1), can be disabling (2), and is related to development of contracture and pain (3, 4). However, longitudinal studies investigating how hand spasticity relates to hand motor recovery, are scarce (4). A better understanding of the relationship between spasticity severity early after stroke and motor recovery could inform about which patients could potentially benefit from different treatment paradigms. For example, spasticity, comprising a velocity dependent increase in tonic stretch reflexes (5), can be reduced by blocking neuromuscular transmission with botulinum toxin. In the upper limb, botulinum toxin has been shown to reduce spasticity and pain and improve limb positioning (6). Results in recent studies also suggest that botulinum toxin treatment has been associated with improvement in both passive and active range of movement post-stroke (7). Further, other non-neural factors (elasticity, viscosity) may also contribute to resistance to passive stretch (8), which may go undetected when using the most frequently applied measure of muscle tone, the modified Ashworth scale (9, 10) and potentially confound clinical trials.

The use of a validated biomechanical assessment (11) could improve diagnostic accuracy by separately measuring the neural component (NC) and non-neural components contributing to passive stretch-resistance (12). Further, improved diagnostic accuracy also opens new opportunities for longitudinal characterization (4, 11), and for charting the relation between severity and lesion location using modern imaging techniques (13). Given that reducing post-stroke spasticity may be associated with improved active range of movement (7) we hypothesized that hand spasticity early post-stroke would relate to poor hand motor recovery. More specifically, we predicted that severe early spasticity would be associated with less longitudinal improvement in clinical measures of sensorimotor hand function. We characterized longitudinal changes in hand spasticity, i.e., the neural component of passive stretch-resistance after stroke, using a validated biomechanical method with normative data, and studied the relation to hand motor recovery, muscle contracture, and pain. We also explored corticospinal tract (CST) lesion load and lesion location using voxel-based lesion symptom mapping (13) to gain insights into the neural correlates of hand spasticity.

## Methods

### Study Design and Participants

**Figure 1:**
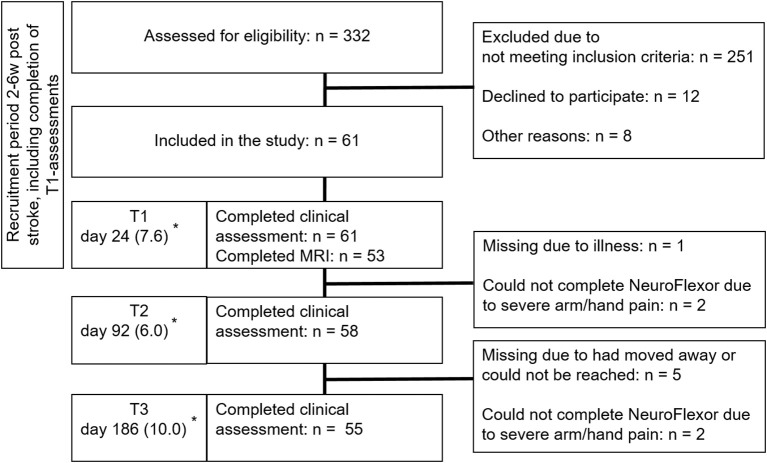
Flow diagram of recruitment of patients. *Number of days from stroke onset to assessment (mean [*SD*]). Reprinted with permission from the Aggeromedtech company (14).

This prospective observational explorative study is part of the ongoing ProHand-study; ClinicalTrials.gov Identifier: NCT02878304. Patients were recruited consecutively from a subacute inpatient rehabilitation unit in Stockholm, Sweden, admitting patients aged 18–67 years from March 2013 until September 2016. In Sweden, patients aged above 67 years are normally referred to geriatric rehabilitation clinics. The study comprised assessments at admission to a subacute in-patient rehabilitation at 2–6 weeks (T1), (on average 3), at 3 (T2) and 6 months (T3) after stroke (Figure 1). Inclusion required: 2–6 weeks after first-ever computed tomography or MRI-verified stroke with upper limb weakness. The admitting team physician verified central paresis of the upper limb at admission by clinical examination. This comprised Manual Muscle Testing and the arm and hand items of the National Institutes of Health Stroke Scale (including finger movements, item 12). Muscle weakness in any muscle group of the upper limb, considered to be stroke related, indicated hemiparesis. Exclusion criteria included inability to understand, comply with instructions, and give informed consent, other disorders affecting hand function, cerebellar lesions or contraindications for MRI. Speech and language therapists participated to optimize the consent process for patients with aphasia. Written informed consent was obtained from all participants. The study was approved by the Regional Ethical Review Board in Stockholm (DNR: 2011/1510-31/3). All procedures were in accordance with the 1975 Declaration of Helsinki.

### Clinical Evaluation

Baseline assessments of global disability included the National Institute of Health Stroke Scale (15), cognitive screening with The Barrow Neurological Institute screen for higher cerebral functions (16), and aphasia with the neurolinguistic instrument Aning (17) and Boston Naming Test (18, 19). Visuospatial attention was assessed using the Baking Tray Task (20) and Albert's test (21) and activities of daily living with the Barthel Index (22). Upper limb sensorimotor impairment, sensation and pain were assessed using the respective domains of the Fugl-Meyer Assessment Upper Extremity (FMA-UE) (23–25). The three reflex-items of the motor domain were excluded (26), yielding maximum 60 points. FMA-HAND (subscale C) evaluated distal sensorimotor impairment (24, 27).

Gross manual dexterity was assessed with the Box and Blocks Test (28, 29), and grip strength with a digital handheld dynamometer (www.seahanmedical.com) (30), taking the mean of three trials. The force of the more affected hand (contralateral to the lesion) was normalized to the less affected hand. Passive range of movement of the wrist with fingers extended, was assessed with a goniometer (31). The fingers were passively positioned in Metacarpophalangeal joints extension at 0 degrees, after which the range of motion of the wrist was measured.

### Resistance to Passive Muscle Stretch

**Figure 2:**
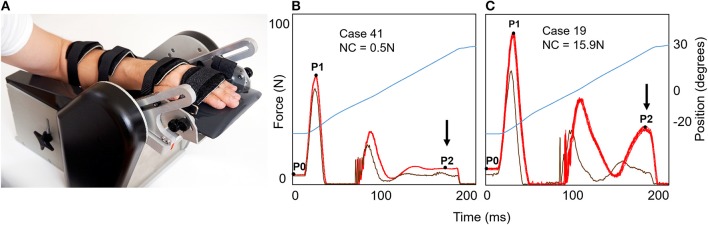
The NeuroFlexor hand module for evaluation of neural and non-neural resistance to passive muscle stretch. **(A)** The NeuroFlexor hand module. The assessment was performed with the patient seated with full back support and the forearm and hand resting on the arm and hand platform. The device was set to passively extend the wrist with a starting angle of 20° palmar flexion to 30° wrist extension at controlled slow (5°/s) and fast (236°/s) velocities. The metacarpophalangeal joints are maintained in slight flexion and the interphalangeal joints in full extension throughout the movement. After one slow and one fast test trials, 5 slow and then 10 fast passive wrist extensions are performed according to standardized protocol (32). Two slow and two fast trials are repeated without the hand to allow subtraction of forces generated by the measurement device. Resistance profiles during 10 fast passive movements (236°/s), force in Newton (N), are shown to the right **(B,C)**. Bright red trace show force generated with hand on platform and dark red trace shows force generated without hand on platform (both necessary to calculate NC). The angle of the hand platform is shown in blue (in total 50° of extension). On the left **(B)**, the patient had minimal increase of late resistance (at P2) during fast stretch (arrow). On the right **(C)**, the patient had a greater increase in late resistance development and this patient had a NC value above cut-off level of 3.4N established in healthy controls (33).

Force components of resistance to passive extension of wrist and finger flexor muscles were measured with the NeuroFlexor method (www.aggeromedtech.com) (Figure 2A). This incorporates a validated biomechanical model, including the subjects body weight (for estimation of inertia), allowing separate calculation of neural (NC), elastic (EC), and viscous (VC) contributions to passive stretch resistance at slow (5°/s) and fast (236°/s) constant velocities (8), thus accounting for velocity dependence in tonic stretch reflexes, proposed a core sign of spasticity by Lance (5). A previous validation study demonstrated a velocity-dependent increase of NC and the corresponding EMG response in the flexor carpi radialis muscle in patients with upper limb spasticity after stroke—but not in controls (8). Further, an ischemic nerve block reduced the stretch reflex and the NC, supporting that NC measures the neural component of the total resisting forces (8). NeuroFlexor measurements have good reliability (inter-rater intraclass correlation coefficient: 0.90–0.94; test-retest: 0.90–0.96) and sensitivity to change (32, 34). The resting tension before the initiation of passive extension is also quantified to ensure that movements were passive. A standardized procedure was followed (see Figure 2A) (32). The less affected hand was examined first followed by the more affected hand (contralateral to the lesion). To distinguish between normal and abnormal force values (see Figures 2B,C), we used normative data established in *n* = 107 healthy subjects based on the mean + 3SD (cut-offs: NC > 3.4 Newton (N), EC > 6.0N, VC > 1.1N and resting tension ≥ 9.0N) (33). Hence, we defined and hereafter refer to hand spasticity as having a NC value above the 3.4N cut-off level. For purposes of comparison, resistance to manually imposed muscle stretch was assessed with modified Ashworth Scale (9).

### Magnetic Resonance Imaging

Imaging was performed with an Ingenia 3.0T MR-system (www.usa.philips.com) with an 8HR head coil. High resolution T1-weighted anatomical images were acquired using TFE 3D (3D gradient echo based sequence): FOV 250 × 250 × 181 mm, matrix 228 × 227, slice thickness 1.2 mm and number of slices 301 (Echo time = shortest, relaxation time = shortest). FLAIR images were acquired using FOV = 250 × 250 × 157 mm, matrix 224 × 224, slice thickness = 1.12 mm, number of slices 280, echo time = 289 ms, repetition time = 4,800 ms, inversion time = 1,650 ms, flip angle = 90.

#### Lesion Maps

Before delineating lesion maps, the T1-weighted images were normalized to MNI template using SPM12 (http://www.fil.ion.ucl.ac.uk/spm/software/spm12/). Cost function masking was used to avoid distortion of lesion by normalization procedure (35). To rule out poor normalization, images were inspected visually. Lesion maps were manually drawn on all axial slices of normalized anatomical images (from T1) using MRIcron (36) by researcher (PL) blinded to all clinical data except the side of the stroke. Lesion location was verified on FLAIR and diffusion images and lesion maps were binarized. A neuroradiologist (EL) and an experienced neurologist (J-CB) verified lesion maps (test of interrater reliability in a subset of 17 lesion maps showed an intraclass correlation = 0.94). Left-sided lesions were flipped to the right to enable group analysis (37). Lesion volume was calculated using MRIcron (http://people.cas.sc.edu/rorden/mricron/index.html).

#### Weighted Corticospinal Tract Lesion Load

The weighted corticospinal tract lesion load (wCST-LL) was calculated by computing the overlap between patient's lesion and the CST template (38), a measure related to motor outcome (39). We used the same method and constructed CST as detailed previously Birchenall et al. (40). In brief, first we constructed this CST template from diffusion weighted imaging (74 directions with b-value of 3,000 s/mm2) in 18 healthy subjects (mean age 31.7 years). Probabilistic tracking was performed using the FMRIB Software Library (FSL 5.06, www.fmrib.ox.ac.uk/fsl) with regions of interest including precentral gyri, posterior limb of internal capsule, cerebral peduncles and anteromedial pons. Resulting CST tracts were normalized into MNI space [see Birchenall et al. (40) for details]. The wCST-LL is expressed as a weighted volume (cc) of lesion-CST overlap.

#### Voxel-Based Lesion Symptom Mapping

Voxel-based lesion symptom mapping was used to study relationships between spasticity and lesion location (the NiiStat toolbox, https://www.nitrc.org/projects/niistat/). We performed two types of voxel-based lesion symptom mapping analysis in 53 patients (with complete NeuroFlexor and MRI data). The Liebermeister test for binomial distributions was used to compare lesion location in patients with NC above (*n* = 30) or below (*n* = 23) cut-off at T3. The Freedman-Lane permutation method was used to investigate lesion location related with NC values at T1 and T3 with FMA-HAND as a nuisance regressor, to control for degree of motor impairment.

### Statistical Analysis

We used Statistica 13.2 (http://statistica.io/latest-version) and IBM Statistica SPSS 23 (https://www.ibm.com/analytics/us/en/technology/spss/) software for the statistical analysis. To explore the clinical significance of spasticity occurrence and severity, spasticity sub-groups were defined according to NC amplitude and temporal pattern. Generalized Expectation Maximization analysis was performed to verify sub-group clusters at T1. Linear Mixed Model for Longitudinal Data was applied to explore longitudinal changes in outcome variables. Results from the best fitting model (with the smallest Akaike Information Criterion) are presented. *Post hoc* tests were adjusted according to Bonferroni. Residuals were plotted to verify that assumptions of the models were met. No imputation of missing data was performed. To control for other stroke-related impairments and demographic data each model was repeated adding the following covariates, one at a time; age, sex, stroke type and side, neglect, National Institutes of Health Stroke Scale, sensation and pain (Table 1 and Table e-1).

**Table 1:** Patient characteristics (*n* = 61) at T1, in mean 24 days from stroke onset.

Age in years, mean (*SD*)	53 (10)
Females, n (%)	20 (30)
Stroke type	
Intracerebral hemorrhage, n (%)	20 (33)
Infarction, n (%)	41 (67)
Right hemisphere, n (%)	37 (61)
Days from stroke onset to assessment, mean (*SD*)	
T1	24 (7.6)
T2	92 (6.0)
T3	186 (10.0)
NIHSS, median (IQR)	6 (3–11)
Barthel Index, median (IQR)	60 (30–97)
Cognitive screening (BNIS), median (IQR)	41 (11–49)
Neglect* n (%)	16 (26)
Aphasia^†^ n (%)	22 (36)
Sensory function^‡^, median (IQR)	5 (0–12)

NIHSS, National Institutes of Health Stroke Scale; BNIS, Barrow Neurological Institute Screen for Higher Cerebral Function (< 47 = cognitive dysfunction).

* Assessed using Baking tray task and Albert's test.

† Assessed using Aning Neurolinguistic aphasia examination, index < 4.7 (range 0–5).

‡ *Assessed using Fugl-Meyer Assessment, subscale H for touch and proprioception (range 0–12)*.

Correlation between NC, outcome measures and lesion size and location were explored using Pearson or Spearman's tests. Multiple regression analysis was applied to investigate partial correlation statistics. Occurrence of pain was analyzed with Fisher's exact test and logistic regression.

For voxel-based lesion symptom mapping we used permutation methods, shown to be useful in controlling for false positives (41). We only studied voxels lesioned in minimum six patients. The significance level, alpha, was set at *p* < 0.05.

## Results

In total, 61 patients were assessed at 3 weeks (Table 1) of whom 53 patients underwent MRI (see Figure 1 for recruitment, attrition and missing data). In two cases NeuroFlexor assessments could not be performed due to passive movement related pain in the hand and fingers, one at T2 only and one patient at both T2 and T3.

**Figure 3:**
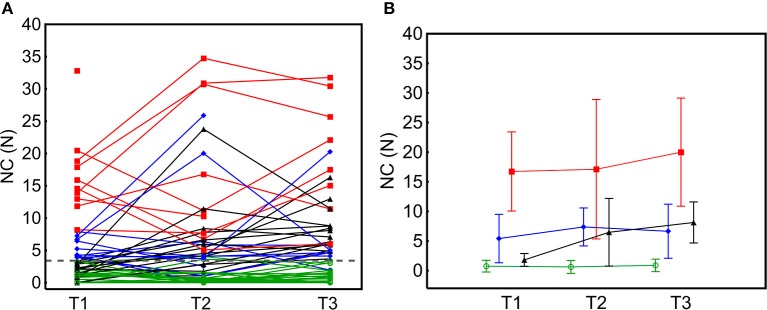
Individual neural component (NC) profiles and group mean across time-points. **(A)** NC over time in individual patients. The individual NC amplitude trajectories were highly variable. **(B)** Mean NC in four different spasticity subgroups identified; No spasticity, NC < 3.4N cut-off in green; Moderate spasticity, NC ≥ 3.4N < 8N in blue; Severe spasticity, NC ≥ 8N in red and Late spasticity, NC ≥ 3.4N in black. Error bars represent 2 standard deviations.

NeuroFlexor assessment showed varying individual levels of NC over time (Figure 3A). Spasticity (NC above cut-off), was present in *n* = 20 (33%) patients at T1, in *n* = 28 (48%) at T2 and in *n* = 28 (51%) at T3. In the cohort as a whole, NC increased over time [T1 to T3: *F*_(2,54)_ = 8.12, *p* = 0.001, Table e-1]. There was no significant interaction effect of Botulinum toxin injection on NC over time [*F*_(2,54)_ = 0.74, *p* = 0.478], but patients who were treated with Botulinum toxin within the study period had a significantly higher level of NC at each time point [main effect *F*_(1,58)_ = 10.5, *p* = 0.002, EM mean difference at T3 = 6.3, standard error = 1.95, CI: 2.4–10.2].

### Spasticity Subgroups

At T1, *n* = 20 patients had hand spasticity (NC range 3.8–32.9N), which was Moderate for *n* = 10 and Severe for *n* = 10 (Figure 3B). The Moderate and Severe hand spasticity groups were defined by the median value (8N) among patients with NC above the 3.4N cut-off at T1. The cluster analysis in patients with spasticity at T1 confirmed qualitative findings (two clusters: NC = 3.92–8.19N and NC = 11.86–32.82N, comparable to Moderate and Severe groups). Of those with no spasticity at T1, *n* = 14 developed Late spasticity and *n* = 27 did not (No spasticity group). Seven patients (in Severe [*n* = 4], Late [*n* = 2] and Moderate [*n* = 1] groups) received botulinum toxin injections on clinical indication in muscles contributing to hand function once during the study period (three patients at more than 10 weeks before the next assessment [T3] and four patients at 2–6 weeks before next assessment [T2 or T3]). Only one patient, who received botulinum toxin injection at 2 weeks before T2, shifted to a lower severity level at next assessment (from Severe to Moderate) while the other six remained in the same spasticity severity level or had shifted to a more severe spasticity level at further assessments.

NeuroFlexor spasticity values and modified Ashworth Scale scores were discordant at T1 with only n = 11 (55%) patients with NC above cut-off showing modified Ashworth Scale ≥ 1 (Table 2). Three patients in the Severe spasticity group had modified Ashworth Scale = 0 at T1. A NC value above cut-off in the, less affected hand was found in *n* = 9 (14%) at T1, in *n* = 7 (12%) at T2, and in *n* = 5 (9%) patients at T3. NC values correlated across hands (R = 0.57, *p* < 0.0001 at T1).

**Table 2:** Number and proportion of patients with hand spasticity assessed with NeuroFlexor and Modified Ashworth Scale.

	**T1**	**T2**	**T3**
Neural Component (NC) > cut-off	20 (0.31)	28 (0.48)	28 (0.51)
Modified Ashworth Scale (MAS) ≥ 1	21 (0.34)	28 (0.48)	30 (0.54)
NC ≥ cut-off and MAS ≥ 1	11 (0.55)	19 (0.68)	22 (0.79)

### Spasticity and Hand Motor Recovery

Early NC, at T1, correlated negatively with FMA-UE, FMA-HAND, passive range of movement, Box and Blocks Test, grip strength and pain (FMA sub-scale) at T3 (range: R = −0.41 to −0.55, *p* ≤ 0.0036, Bonferroni corrected) (Table 3).

**Table 3:** Association between clinical assessments of sensorimotor disability at each time-point and NeuroFlexor assessments of spasticity (NC) at T1.

	**T1**	**T2**	**T3**
NC with FMA-UE*	−0.34	−0.30	−0.51**
NC with FMA-HAND^†^	−0.42**	−0.37**	−0.55**
NC with Sensory function^‡^	−0.12	−0.18	−0.21
NC with Grip strength	−0.42**	−0.39	−0.46**
NC with Box and Block Test	−0.52**	−0.39**	−0.54**
NC with ROM^§^	−0.29	−0.25	−0.45**
NC with FMA pain^||^	−0.12	−0.28	−0.41**

Numbers are Spearman's rho.

** Significant after Bonferroni correction (p ≤ 0.0036).

* FMA-UE, Fugl-Meyer Assessment for Upper Extremity;

† FMA-HAND, Fugl-Meyer Assessment HAND sub-scale;

‡ FMA sub-scale H for somatosensory function (0–12 points);

§ ROM, Passive Range of Movement of the wrist, fingers extended;

|| *FMA subscale for pain during passive movement (0–24 points, higher score equals less pain)*.

**Figure 4:**
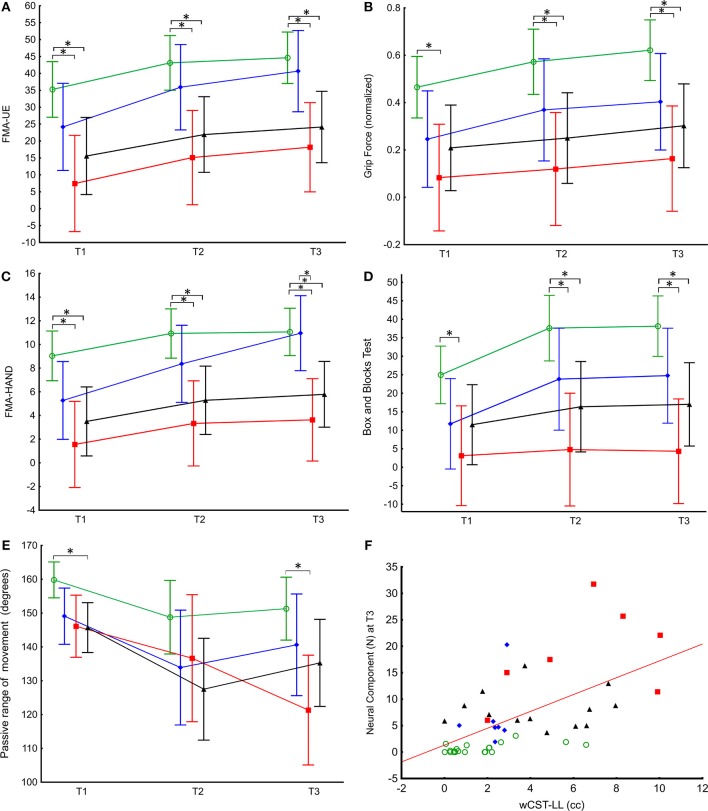
Active and passive hand function across time in relation to spasticity subgroups and linear association between NC amplitude and weighted CST lesion load. **(A)** FMA-UE and maximal grip strength **(B)** showed similar recovery pattern over time in the four spasticity subgroups; Severe spasticity (red), Moderate spasticity (blue), No spasticity (green), and Late developing spasticity (black). Estimated marginal means and 95% confidence intervals (linear mixed effects model) are shown. **(C)** The Moderate spasticity group (blue) had significantly larger change in FMA-HAND than the other three spasticity groups. **(D)** There was a significant increase in number of blocks transferred (Box and Blocks Test score) from T1 to T3 in the No spasticity and Moderate spasticity groups (see also Table e-1). **(E)** Severe spasticity was the only group with continued decrease in passive range of movement from T2 to T3 (see also Table e-1). **(F)** Relationship between wCST-LL and NC at T3. Color coding for spasticity subgroup comparisons. *p < 0.05.

Hand motor recovery differed in the four spasticity subgroups (Table e-1 and Figures 4A–E). Concerning FMA-UE, there was a significant main effect of time [*F*_(2,60)_ = 28.6, *p* < 0.0001] and group [*F*_(3,60)_ = 6.2, *p* = 0.0001] but no significant group-by-time interaction [*F*_(6,60)_ = 0.9, *p* = 0.507] (Figure 4A). Maximal grip strength recovered similarly over time in all groups [main time effect: *F*_(2,54)_ = 11.8, *p* < 0.0001; group: *F*_(3,57)_ = 5.0, *p* = 0.004; group-by-time: *F*_(6,54)_ = 0.77, *p* = 0.597] (Figure 4B). Box and Blocks Task scores also improved over time [*F*_(2,56)_ = 8.0, *p* = 0.001] and subgroups differed [*F*_(2,58)_ = 8.0, *p* = 0.018] and there was no group-by-time interaction [*F*_(4,57)_ = 1.74, *p* = 0.378]. Only the No spasticity and Moderate spasticity groups increased significantly over time (Table e-1 and Figure 4D). FMA-HAND showed a significant main effect of time [*F*_(2,58)_ = 22.4, *p* < 0.0001] and group [*F*_(2,58)_ = 3.94, *p* < 0.0001], and a significant group-by-time interaction [*F*_(4,60)_ = 5.9, *p* < 0.001] (Figure 4C). *Post hoc* comparisons showed significant differences between No spasticity and both Severe and Late spasticity groups at all-time points and between the Moderate and Severe spasticity groups at T3. All except the Severe spasticity group improved significantly to T3 (Table e-1, Figure 4C).

There was a significant decrease in passive range of movement over time [*F*_(2,59)_ = 9.3, *p* < 0.001], with a group effect [*F*_(2,58)_ = 8.0, *p* = 0.018] and a significant group-by-time interaction [*F*_(4,59)_ = 5.25, *p* = 0.001] (Figure 4E). *Post hoc* comparisons revealed significant differences in passive range of movement between No spasticity and Severe spasticity groups at T1 and T3. Only the Severe spasticity group decreased significantly to T3 (Table e-1).

The statistically significant mixed model effects of spasticity group-by-time interaction on hand motor recovery remained also when controlling for demographic and stroke-related impairment variables (age, sex, stroke type and side, neglect, National Institutes of Health Stroke Scale, sensation and pain) as well as botulinum toxin injection. The results also remained significant after excluding the four patient who received Botulinum toxin injections within 6 weeks before 3 or 6 months follow up assessments.

The Severe spasticity group had a larger proportion of patients reporting pain at T3 (Fischer exact: *p* = 0.028, Table e-2). Based on the odds ratio, the odds of having pain at T3 was 9.1 (95% CI: 1.05–78.54) times higher for the Severe spasticity group compared to No spasticity patients.

### Spasticity and Lesion Size and Location

The wCST-LL varied widely between patients (mean = 2.2, *SD* = 2.6, range 0–14.6). wCST-LL correlated with NC at T1 (R = 0.49, *p* = 0.0004) and T3 (R = 0.61, *p* < 0.0001) (Figure 4F). Mixed model analysis of NC over time revealed a main effect of wCST-LL [*F*_(1,38)_ = 18.7, *p* = 0.0001] but no statistically significant interaction. Multiple regression analysis including FMA-HAND, wCST-LL, and lesion volume showed that only wCST-LL predicted NC at T1, T2 and T3 Partial correlation at T1: R = 0.34, *p* = 0.022; T2: R = 0.32, *p* = 0.048 and T3: R = 0.43, *p* = 0.005, Table 4).

**Table 4:** Multiple regression analyses of Neural Component (NC) at T1, T2 and T3 with FMA-Hand sub-scale, wCST-LL, and lesion volume as predictors.

**Outcome**	**Predictors**	**VIF**	**Unstandardized coefficients**		**Standardized coefficients**		**95% Confidence intervals**		***t***	**Sig**.
			**B**	**Std. Error**	**Beta (β)**	**Std. Error (β)**	**Lower**	**Upper**		
Neural Component at T1	(Constant)		3.11	1.95					1.59	0.118
	FMA-HAND	1.4	−0.18	0.17	−0.16	0.16	−0.47	0.15	−1.04	0.303
	wCST–LL	1.6	0.97	0.40	0.39	0.17	0.06	0.73	2.37	0.022
	Lesion Volume	1.4	0.001	0.01	0.05	0.16	−0.26	0.37	0.33	0.743
Neural Component at T3	(Constant)	5.15	3.65						1.41	0.167
	FMA-HAND	−0.29	0.25						−1.15	0.253
	wCST-LL	1.68	0.82						2.05	0.0477
	Lesion Volume	−0.01	0.02						−0.51	0.612
Neural Component at T3	(Constant)		5.06	2.48					2.04	0.047
	FMA-HAND	1.6	−0.31	0.21	−0.24	0.16	−0.56	0.08	−1.54	0.135
	wCST-LL	1.8	2.08	0.70	0.50	0.17	0.16	0.84	2.97	0.005
	Lesion Volume	1.3	−0.01	0.02	−0.07	0.14	−0.36	0.22	−0.48	0.631

*VIF, Variance Inflation Factor.

**Figure 5:**
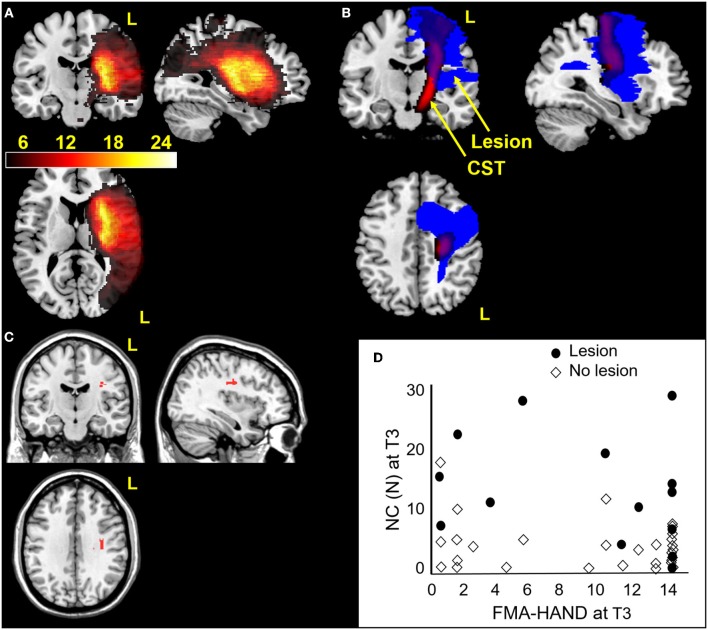
Hand spasticity in relation to MRI measures of cerebral lesion location. **(A)** Lesion locations in 53 stroke patients overlaid on coronal, sagittal and axial slices of template normalized T1-weighted image. Lesions showed greatest overlap in middle cerebral artery territory. **(B)** Corticospinal tract template (red) and stroke lesion (blue) in a patient with a large lesion (17cc). The patient had severe impairment (FMA-HAND = 0). **(C)** Voxel based lesion symptom mapping results showing significant voxels (in red) relating to increased NC values at T3 (*p* < 0.05, FDR corrected), controlling for FMA-HAND. **(D)** NC and FMA-HAND values at T3 plotted for patients with lesions in significant voxels found in Voxel based lesion symptom mapping analysis (shown in red [C]). Note that higher NC values were present in patients with lesions in the subcortical white matter beneath the hand knob [C] and this was not related to FMA-HAND scores (e.g., high NC was also found in patients with maximal FMA-HAND score).

In all patients, lesion volume correlated with NC at T1 (R = 0.37, *p* = 0.016), but not at T3 (R = 0.27, *p* = 0.084; Figure 5A). Higher wCST-LL values were detected in patients with greater motor impairment (Figure 5B).

Voxel-based lesion symptom mapping analysis showed that NC at T3 was significantly related to lesion in white matter underneath the cortical “hand knob” (28), within the CST. This cluster of 54 voxels remained significant when including FMA-HAND at T3 as nuisance variable (Freedman-Lane permutation, Figure 5C). A lesion in this area explained unique variance in NC since high FMA-HAND values were equally distributed in patients with or without lesions in this area (Figure 5D). Binomial Voxel-based lesion symptom mapping analysis showed an association with lesion in the same brain location with NC > cut-off.

### Mechanical Contributions to Passive Muscle Stretch Resistance

EC was above the 6.0N cut-off in *n* = 2 patients, (3%) at T1, *n* = 5 (9%) at T2 and in *n* = 7 (13%) at T3. EC increased significantly over time in the Severe spasticity group only (F_2,54_ = 3.8, *p* = 0.028) (Table e-1). NC, EC and VC did not correlate. EC correlated with passive range of movement at T1 (R = −0.30, *p* = 0.027), T2 (R = −0.51, *p* < 0.0001) and T3 (R = −0.48, *p* = 0.0003). Many patients also showed increased VC although this only contributed about 14% to the total average passive movement resistance at T1 and did not change over time (e-1).

## Discussion

This study provides longitudinal data on hand spasticity after stroke and its relation to hand sensorimotor recovery and development of secondary complications. A strong association between degree of hand spasticity and lesion of the CST was also demonstrated. These findings and their clinical implications are discussed below.

### Hand Spasticity Severity in Relation to Motor Recovery and Development of Contracture and Pain

Hand spasticity occurred in up to half of the patients studied, comparable to previous reports on upper limb spasticity (1, 3, 42, 43), using manual tests. Three spasticity subgroups were apparent. Early spasticity, present at 3 weeks after stroke, was either Moderate (NC = 3.4–8 N) or Severe (> 8 N) and a third group developed spasticity more gradually by 3 or 6 months (Late spasticity). The majority of patients showed stable or increasing spasticity over time (Figure 3). This is in line with previous reports using biomechanical measures (4, 11), and does not support the view that spasticity declines over time in parallel with motor recovery (44).

Spasticity subgroups showed different degrees of hand motor recovery, development of muscle contracture and pain over time. The Severe group, representing 16% of studied patients, showed negligible improvement in FMA-HAND (non-significant difference over time of two points) compared to the Moderate group (significant difference over time of 6 points), despite overlapping scores at T1 (Figure 4C). Both the Severe and Late spasticity groups also failed to show significant improvement in Box and Blocks scores over time (Figure 4D). These findings suggest that early severe and late developing hand spasticity can impair recovery of voluntary finger movements (FMA-HAND) and grasp and release function (Box and Blocks Test). Although weakness (45) and degree of lesion to the CST (39), or a combination thereof (46), are the major predictors of hand motor recovery, our findings indicate a certain contribution of spasticity. This is supported by findings showing enhanced velocity and smoothness of upper limb movements after anti-spastic botulinum toxin injections in the chronic phase after stroke (47) and by findings by de Gooijer-van de Groep et al. who showed that reflexive torque at the wrist was higher in stroke patients with poor motor recovery (indicated by reduced finger extension and shoulder abduction strength) (48). However, the degree of spasticity is also important to consider, since patients with Moderate spasticity showed equal or greater hand motor recovery over time compared to patients without hand spasticity.

Early Severe hand spasticity was also associated with development of contractures and pain as previously suggested (3, 4). Hand spasticity preceded muscle contracture: only 3% of the patients had elastic resistance (EC) above norm-based cut-off at T1. Furthermore, no differences in resting tension were found in stroke patients (49). The results show that spasticity (NC) is the major contributor to passive movement resistance in both the early and chronic phase after stroke (8, 33, 34), contradicting some previous findings (50).

### Corticospinal Tract and Spasticity

This study provides the first data showing that hand spasticity is related to lesion load of the CST, even when controlling for motor impairment and lesion volume. However, some patients with Severe and without hand spasticity (No spasticity) had similar CST lesion load (Figure 5D), suggesting that lesion to certain parts of the CST may have a specific role in the development of hand spasticity. This hypothesis was supported by the voxel-based lesion symptom mapping results, which identified a region in the subcortical white matter below both the pre- and postcentral gyri (Figure 5C), where the largest tract passing is the CST. Small lesions confined to the CST can lead to spasticity (51). We did not find a relation to lesion location in the basal ganglia (52), although a number of patients had basal ganglia lesions (Figure 5A). The CST is involved in modulating spinal reflexes and sensory gain (53). Recent neurophysiological studies also suggest a key role of the CST in disturbed modulation of spinal reflex activity in patients with spasticity (54, 55). Although our results are in accord with these observations, future studies coupling CST lesion load and neurophysiological measures of spinal circuitry would allow further insight into this question (56).

### Clinical Implications

The NeuroFlexor assessments allowed detection of early hand spasticity in patients with a modified Ashworth (33, 57) score of zero, i.e., with no signs of spasticity at manual testing. We also observed increased NC in the ipsilesional, less affected hand, which has been reported but is often overlooked (58). Early quantitative detection of severe hand spasticity would allow targeted anti-spastic treatment with botulinum toxin (59). Continued monitoring of hand spasticity after the early phase is also indicated to detect late developing spasticity. Treating patients with severe or late developing hand spasticity may promote recovery of volitional hand and finger movements and limit development of contractures and pain.

Notably, although this study found a strong association between early spasticity and recovery of motor function, the study does not inform on how spasticity relates to active motor performance, which has long been a matter of debate (60). However, the results have implications for prediction of hand spasticity and of degree of motor recovery after stroke. Spasticity was associated with hand motor recovery and large CST lesions (lesion load >7 cc, Figure 5D), especially those lesions covering white matter below the hand knob (61), predicted more severe spasticity at 6 months after stroke.

## Limitations

The NeuroFlexor method enables standardized and clinically feasible quantification of force resistance of neural and non-neural origin. It should be pointed out that the NeuroFlexor method has some recognized limitations and does not include EMG recordings (62). However, regarding its demonstrated validity (8), we consider this method superior to manual assessments and a relevant alternative to more demanding experimental set ups when performing the current type of clinical studies.

The number of patients within subgroups was relatively small (*n* = 10 in the Moderate and Severe spasticity groups). However, the significant results suggest an adequate sample size even though we cannot rule out that a larger sample could have changed results to some degree. Another issue is that we only included patients aged below 70 years, referred to subacute in-patient rehabilitation. We cannot rule out that patients with differing stroke severity and not represented in this sample, have different spasticity development. Finally, patients were followed until 6 months after stroke. Although motor recovery is most pronounced in the first months after stroke, spasticity, muscle contractures and pain may develop later.

## Conclusions

Finger and wrist flexor muscle spasticity was common with increasing occurrence and severity over time. Patients with early severe hand spasticity showed poorer hand motor recovery, decreased passive range of movement and reported arm pain more frequently over time. This contrasted with findings from the no and moderate hand spasticity groups who showed greater hand motor recovery. Hand spasticity was related to lesion of the CST, independently of total lesion volume and initial hand motor impairment. A lesion in the white matter below the hand knob was related to more severe hand spasticity at 6 months. This study advances our knowledge regarding the development of hand spasticity after stroke and findings are relevant for development of targeted treatment approaches and for the prediction of hand motor recovery post stroke.

## Data Availability

The datasets for this manuscript can be made available on reasonable request but are not made public since this study make part of a larger data collection that is under ongoing analysis. Requests to access the data should be directed to jeanette.plantin@ki.se.

## Author Contributions

JP, EL, JB, and PL: study concept and design and study supervision was performed. JP, GP, and KW: acquisition of data was performed. JP, PR, J-CB, AG, and PL: analysis and interpretation of data was performed. JP, JB, GP, PR, J-CB, KW, AG, and PL: critical revision of manuscript for intellectual content was performed.

### Conflict of Interest Statement

The NeuroFlexor instrument described in this paper has been patented and the author PL is a shareholder in the manufacturing company Aggero MedTech AB. The remaining authors declare that the research was conducted in the absence of any commercial or financial relationships that could be construed as a potential conflict of interest.
