# Supplementary material for: Quantitative Assessment of Hand Spasticity After Stroke: Imaging Correlates and Impact on Motor Recovery
Source: Front Neurol. 2019 Aug 12;10:836. doi: 10.3389/fneur.2019.00836 (PMC6699580; doi:10.3389/fneur.2019.00836)
Supplement: Supplementary file 1 [file Data_Sheet_1.PDF]

Supplementary Material - Spasticity quantification and motor recovery

e-1 Supplementary Material - Results

Table e-1 Estimated Marginal (EM) means at 3 weeks, 3 and 6 months and difference in EM means over time.

Hand function and activity measures by spasticity sub-groups		EM means (SE)			Difference in EM means over time			
		T1	T2	T3	T1 minus T3	95% CI		*p v*alue
NC*	All patients	4.38 (0.81)	6.12 (1.20)	7.05 (1.14)	-2.67	(-4.30	-1.05)	<0.001
	No	0.75 (0.45)	1.30 (1.20)	0.93 (0.97)	-0.18	(-2.19	1.82)	1.000
	Moderate	5.66 (0.76)	7.40 (1.98)	8.04 (1.65)	-2.38	(-5.88	1.12)	0.297
	Severe	17.70 (0.84)	20.76 (2.29)	22.12 (1.33)	-4.42	(-8.24	-0.60)	0.018
	Late	1.80 (0.68)	6.48 (1.76)	8.12 (1.34)	-6.32	(-9.07	-3.57)	<0.001
EC†	All patients	3.53 (0.18)	3.92 (0.23)	3.84 (0.25)	0.32	(-0.89	0.25)	0.517
	No	3.70 (0.26)	3.76 (0.34)	3.49 (0.33)	0.20	(-0.56	0.97)	1.000
	Moderate	3.06 (0.41)	3.68 (0.51)	3.33 (0.58)	-0.27	(-1.60	1.07)	1.000
	Severe	3.55 (0.46)	4.22 (0.59)	5.17 (0.62)	-1.62	(-3.07	-0.72)	0.023
	Late	3.80 (0.37)	4.00 (0.46)	3.38 (0.40)	-0.41	(-0.64	1.46)	0.998
VC†	All patients	1.22 (0.08)	1.20 (0.09)	1.10 (0.09)	0.12	(-0.10	0.34)	0.544
	No	1.11 (0.12)	1.01 (0.13)	0.97 (0.13)	0.14	(-0.18	0.46)	0.852
	Moderate	1.12 (0.19)	1.21 (0.20)	1.14 (0.23)	0.06	(-0.51	0.62)	1.000
	Severe	1.56 (0.21)	1.52 (0.23)	1.35 (0.25)	0.21	(-0.40	0.82)	1.000
	Late	1.23 (0.17)	1.34 (0.18)	1.17 (0.18)	0.06	(-0.38	0.50)	1.000
FMA-UE §								
	All patients	20.61 (2.87)	29.01 (2.81)	31.90 (2.66)	-11.28	(-14.96	-7.61)	<0.001
	No	35.26 (3.97)	43.10 (3.89)	44.61 (3.67)	-9.35	(-14.39	-4.32)	<0.001
	Moderate	24.18 (6.22)	35.91 (6.09)	40.67 (5.80)	-16.49	(-24.56	-8.42)	<0.001
	Severe	7.44 (6.87)	15.11 (6.73)	18.17 (6.37)	-10.72	(-19.05	-1.95)	0.011
	Late	15.57 (5.51)	21.93 (5.40)	24.14 (5.09)	-8.57	(-15.54	-1.60)	0.011
FMA-HAND ǀǀ								
	All patients	4.84 (0.73)	6.96 (0.73)	7.89 (0.70)	-3.05	(-4.17	-1.93)	<0.001
	No	9.04 (1.01)	10.93 (1.00)	11.06 (0.96)	-2.02	(-3.56	-0.49)	0.006
	Moderate	5.27 (1.59)	8.36 (1.57)	10.95 (1.53)	-5.68	(-8.14	-3.22)	<0.001

Severe	1.56 (1.75)	3.33 (1.74)	3.75 (1.68)	-2.20	(-4.87	0.48)	0.142
Late	3.50 (1.41)	5.21 (1.39)	5.79 (1.34)	-2.29	(-4.41	-0.16)	0.031
		FMA-HAND Pairwise comparisons of difference in EM mean (No minus Moderate etc.)					
		No	Moderate	-3.66	(-6.01	-1.30)	0.003
		No	Severe	-0.18	(-2.68	2.33)	0.889
		No	Late	-0.27	(-2.39	1.87)	0.806
		Moderate	Severe	3.48	(0.53	6.43)	0.021
		Late	Moderate	-3.40	(-6.04	-0.76)	0.013
		Late	Severe	0.09	(-2.69	2.86)	0.950
Grip Strength							
All patients	0.21 (0.05)	0.25 (0.03)	0.30 (0.09)	-0.09	(-0.19	-0.01)	0.059
No	0.46(0.06)	0.57 (0.07)	0.62 (0.06)	-0.16	(-0.24	-0.07)	<0.001
Moderate	0.25 (0.10)	0.37 (0.11)	0.40 (0.10)	-0.16	(-0.30	-0.01)	0.027
Severe	0.083 (0.11)	0.12 (0.12)	0.16 (0.11)	-0.08	(-0.23	0.07)	0.600
Late	0.21 (0.09)	0.25 (0.10)	0.30 (0.09)	-0.09	(-0.21	0.03)	0.178
Box and Blocks Test							
All patients	12.82 (2.72	20.64 (3.08)	21.05 (2.85)	-8.23	(-12.78	-3.67)	<0.001
No	24.96 (3.76)	37.60 (4.28)	38.14 (3.94)	-13.18	(-19.48	-6.87)	<0.001
Moderate	11.73 (5.89)	23.82 (6.65)	24.76 (6.20)	-13.03	(-22.99	-3.06)	0.006
Severe	3.11 (6.51)	4.78 (7.35)	4.32 (6.82)	-1.21	(-12.10	9.69)	1.000
Late	11.50 (5.22)	16.36 (5.90)	17.00 (5.43)	-5.50	(-14.11	3.11)	0.362
ROM #							
All patients	150.18 (1.85)	136.71 (3.79)	137.14 (3.29)	13.05	(6.16	19.93)	0.001
No	159.82(2.56)	148.78 (5.25)	151.31 (4.49)	8.50	(0.86	17.87)	0.087
Moderate	149.09 (4.01)	133.91 (8.19)	140.62 (7.26)	8.47	(-6.83	23.77)	0.536
Severe	146.11 (4.44)	136.67 (9.06)	121.33 (7.84)	24.78	(8.38	41.19)	0.001
Late	145.71 (3.56)	127.50 (7.26)	135.29 (6.21)	10.43	(-2.52	23.37)	0.155
		ROM Pairwise comparisons of mean difference (No minus Moderate etc.)					
		No	Moderate	0.04	(-14.54	14.62)	0.996
		No	Severe	-16.28	(-31.62	-0.94)	0.038
		No	Late	-1.92	(-14.90	11.05)	0.768
		Moderate	Severe	-16.32	(-34.54	1.91)	0.079
		Late	Moderate	1.96	(-14.32	18.24)	0.811
		Late	Severe	-14.36	(-31.33	2.62)	0.096

* NeuroFlexor measures of neural (NC) and † mechanical resistance (EC and VC). ‡ FMA-UE - Fugl-Meyer Assessment for Upper Extremity (0-60 points, 3 reflex items excluded), § FMA-HAND – Fugl-Meyer Assessment HAND sub-scale (0-14 points), | | ROM – Passive Range of Movement of the wrist, fingers extended. Estimates are corrected according to Bonferroni.

e-1 Mechanical contributions to resistance to passive muscle stretch

Elasticity (EC) was above the 6.0N cut-off in n = 2 patients (3.3%) at T1, n = 5 patients (8.6%) at T2 and in n = 7 patients (12.7%) at T3. In all patients, mean (SD) EC at T1 was 3.58 (1.37) N, 3.84 (1.65) N at T2 and 3.62 (1.76) N at T3.

Only the Severe spasticity group had a significant increase in EC over time ($F_{2, 54} = 3.8$, $p = 0.028$) (table e-1). Viscosity (VC) was above the 1.1N cut-off in 50.8% of patients at T1, in 51.7% at T2 and in 47.3% at T3. In all patients, mean (SD) VC was 1.20N (0.64) at T1, 1.22N (0.69) at T2 and 1.08N (0.71) at T3. No significant time or group effect was found.

At T1, n = 1 patient had a resting tension value above the 9.0N cut-off, n = 2 patients at T2 and no patient at T3.

After correction for multiple comparisons, Pearson´s correlation between NC, EC and VC did not yield significant results. EC correlated negatively with Range of Movement at T1 ($R = -0.30$, $p = 0.03$), T2 ($R = -0.51$, $p < 0.0001$) and T3 ($R = -0.48$, $p = 0.0003$).

Table e-2 Proportion of arm pain in the respective spasticity subgroup at each time of assessment

	No Spasticity	Moderate Spasticity	Severe Spasticity	Late Spasticity
T1	0.30	0.40	0.50	0.57
T2	0.46	0.60	0.70	0.71
T3	0.35	0.43	0.89	0.71

Arm pain was assessed using the Fugl-Meyer sub-scale in which passive movements are performed by the examiner in 12 upper limb joints. Having arm pain was defined as having ≤ 23 points out of 24, i.e. at least one movement was reported by the patient as with some or pronounced pain.
